# The prevalence of underweight, overweight and obesity in Bangladeshi adults: Data from a national survey

**DOI:** 10.1371/journal.pone.0177395

**Published:** 2017-05-16

**Authors:** Tuhin Biswas, Sarah P. Garnett, Sonia Pervin, Lal B. Rawal

**Affiliations:** 1 Health Systems and Population Studies Division, icddr,b, 68 Shaheed Tajuddin Ahmed Sharani, Mohakhali, 22 Dhaka, Bangladesh; 2 The Children's Hospital at Westmead Clinical School, University of Sydney, Sydney, Australia; 3 Institute of Endocrinology and Diabetes, The Children's Hospital at Westmead, Australia, Westmead, NSW, Australia; 4 James P Grant School of Public Health, BRAC University, Dhaka, Bangladesh; RTI International, UNITED STATES

## Abstract

**Background:**

Over the two last decades Bangladesh, a low-income country, has experienced a rapid demographic and epidemiological transition. The population has increased substantially with rapid urbanization and changing pattern of disease, which at least in part, can be explained by nutritional changes. However, the nutritional status of the adult population has not been previously described. Hence, the objective of this study was to estimate the prevalence and explore socio-demographic determinants of underweight, overweight and obesity among the Bangladeshi adult population.

**Methods:**

This study is a secondary data analysis of the national 2011 Bangladesh Demographic and Health Survey. We determined the nutritional status of adults aged ≥35 years of age, who had a measured weight and height, using the Asian body mass index (BMI) cut-offs for underweight (BMI <18.5 kg/m^2^), overweight (BMI 23 to <27.5 kg/m^2^) and obesity (BMI ≥27.5 kg/m^2^). Logistic regression modeling was used to determine the association between socio-demographic factors and nutritional status.

**Result:**

Of total sample (n = 5495), 30.4% were underweight, 18.9% were overweight and 4.6% were obese. Underweight was associated with age, education and wealth. The adjusted odd ratios for underweight were higher for older people (≥70 years) compared to younger, the least educated compared to the most educated and the poorest compared to the wealthiest were 2.51 (95%CI: 1.95–3.23, p<0.001), 3.59 (95%CI: 2.30–5.61, p<0.001) and 3.70 (95%CI: 2.76–4.96, p<0.001), respectively. Younger age (35–44 years), being female, higher education, wealthier and living in urban areas were associated with overweight/obesity with adjusted odds ratios of 1.73 (95%CI: 1.24–2.41, p<0.001), 2.48 (95%CI: 1.87–3.28, p<0.001), 3.98 (95%CI: 2.96–5.33, p<0.001), 7.14 (95%CI: 5.20–9.81, p<0.001) 1.27 (95%CI: 1.05–1.55, p-0.02), respectively.

**Conclusion:**

Underweight and overweight/obesity are prevalent in Bangladeshi adults. Both conditions are associated with increased morbidity and mortality and increase the risk of developing non-communicable diseases. Effective public health intervention approaches are necessary to address both these conditions.

## Background

Due to nutritional transition, many developing countries are experiencing a shift from underweight to overweight [1]. In 2015, the United Nations Food and Agriculture Organization estimated that 795 million people of the 7.3 billion people worldwide were suffering from chronic under nutrition [2]. Almost all (780 million) lived in developing countries, representing ~13% of the population of developing counties [2]. The consequences of under nutrition in adulthood have been well described, including increased morbidity and mortality, reduced performance capacity and low productivity and for women, adverse pregnancy outcomes [3]. However, over the past two decades, there has been an increasing trend in overweight and obesity in developing countries alongside undernutrition. Overweight and obesity are major determinants of chronic illness, including cardiovascular disease, diabetes, musculoskeletal disorders and some types of cancers [4, 5]. The increasing numbers of people who are overweight or obese are contributing to the growing burden of non-communicable diseases in developing countries [6, 7]. Globally, 3.4 million deaths were estimated to be attributable to overweight and obesity in 2010 [8].

Over the last two decades, Bangladesh, a low-income country, has experienced a rapid demographic and epidemiological transition [9, 10]. The population is projected to increase substantially from 147 million in 2007 to 218 million in 2050 [11]. The population change has been associated with rapid urbanization and a changing pattern of disease [11, 12]. In 1986, the total deaths due to chronic diseases was 8% which increased to 68% by 2006 in rural Bangladesh [13]. At least in part, this can be explained by changes in nutritional status. A recent study of married women in Bangladesh indicated that the prevalence of underweight, normal weight, and overweight was 24.1%, 46.7%, and 29.2% respectively—reflecting the problem of both under and overweight [14]. However, nationally representative data on underweight, overweight and obesity in the adult population in Bangladesh has not been previously published. Hence, we present the prevalence and socio-demographic determinants of underweight, overweight and obesity among the adult population aged ≥35 years, using the nationally representative data from the Bangladesh Demography and Health Survey (BDHS), 2011.

## Methods

### Study design

**Fig 1:**
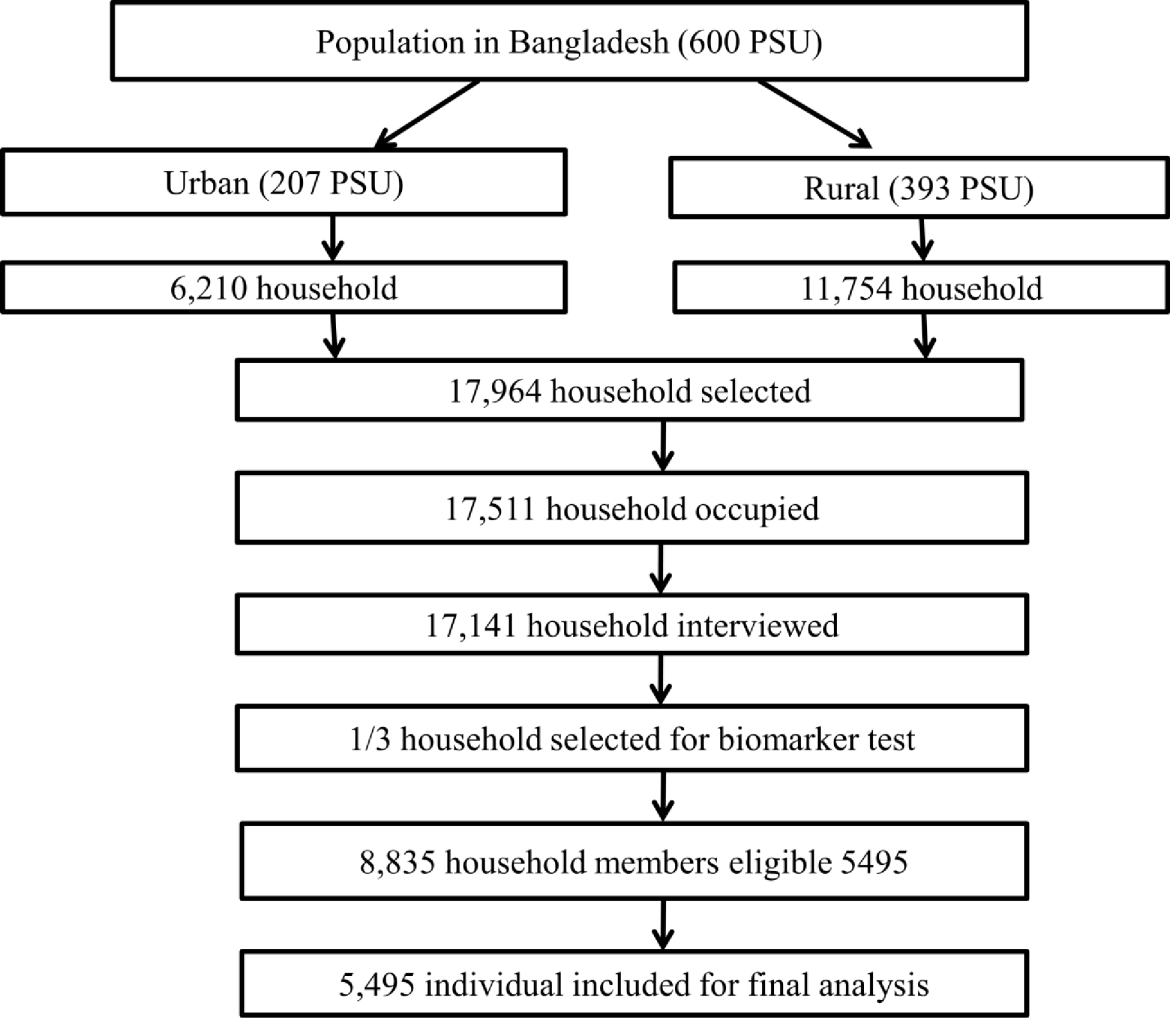
Sampling design of primary data collection.

This study is secondary data analysis of the BDHS, 2011. The BDHS 2011 is a cross-sectional, national representative survey conducted between July and December 2011 and was collaboration between the National Institute of Population Research and Training (NIPORT), ICF International (USA), and Mitra and Associates. The participants in the BDHS were selected using probability sampling based on a two-stage cluster sample of households, stratified by rural and urban areas in the seven administrative regions of Bangladesh. The detailed protocol and methods have been published previously [15]. In brief, 17,500 households were surveyed, of which one in three households were randomly selected for the residents to have anthropometric measurements. This sub-sample included 8835 (4524 men) participants aged ≥35 years. The analysis presented in this manuscript is based on adults ≥35 years of age who had anthropometry measured (n = 5640) of which 145 had missing data(**Fig 1).**

### Anthropometry measurements

Weight and height were measured at the participant’s home by trained field research staff. Weight was measured twice to the nearest 0.1 kg with light clothing on and without shoes by digital weighing scales placed on a flat surface. The average of the two measurements was used in the analysis. Height was measured three times using a standard clinical height scale with patient standing without shoes. The average of the measurements was used in the analysis. BMI was calculated as weight (kg)/height (m^2^). Asian specific BMI cut-offs were used to define underweight (<18.5 kg/m^2^), overweight (23.0 to <27.5 kg/m^2^) and obese (≥27.5 kg/m^2^) [16].

Age and sex as well as demographic characteristics, including educational status, involvement in paid work, and region (district) and place (urban or rural) of residence was collected by questionnaire which was administered during a face to face interview. Household wealth index is a composite measure of a household's cumulative living standard. The index is calculated using household's ownership of selected assets, including electricity, televisions and bicycles; materials used for housing construction; types of water access and sanitation facilities; use of health and other services, and in health outcomes. It is determined using principle components analysis. National-level wealth quintiles (from lowest to highest) are obtained by assigning the household score to each de jure household member, ranking each person in the population by his or her score, and then divided the ranking into five quintile, each comprising 20 percent of the population [17]. [18, 19]. Ethics approval for BDHS, 2011 was obtained from the Medical Research Council of Bangladesh and informed consent was given by the participants.

### Statistical analyses

The study considers socio-demographic factors to assess the determinants of underweight and overweight of the study participants. The effect of one variable on the prevalence of undernutrition is likely to be confounded with the possible effects of other variables. Therefore, socio-demographic characteristics were controlled statistically. The variables included as covariates were: age at interview, sex, education status, number of family members in the household, current work status, wealth index, administrative region and place of residence (urban or rural). The weighted prevalence of underweight, normal weight, overweight and obesity was obtained for each category using the national weights assigned by the cluster design at the primary sampling unit level. The chi-square tests were used to identify differences in proportions of BMIs between the categories of the exposure and outcome variables. To assess the net effect of exposure variables on the outcome measures logistic regression analysis was conducted. All analyses, univariate and multivariate logistic regression, were adjusted for cluster and sample weight and completed in SPSS (IBM, 21).

## Results

The socio-demographic characteristics of the 5495 participants are presented in Table 1.

**Table 1:** Socio-demographic characteristics participants (n = 5495), stratified by nutritional status. Data are presented as percentages and 95% CI.

Variables	Underweight (<18.5 kg/m2)	Normal weight (18.5 to <23.5 kg/m2)	Overweight/obese (≥23 kg/m2)
**Age group**			
35–44	21.2 (18.7–24.1)	53.8 (50.8–56.7)	25.0 (22.2–27.9)
45–54	29.0 (26.1–32)	47.0 (44.1–49.8)	24.1 (21.6–26.7)
55–69	32.9 (30.2–35.7)	46.7 (44.1–49.3)	20.4 (18.1–22.9)
70+	46.5 (42.7–50.4)	38.6 (35.1–42.2)	14.9 (12.4–17.8)
**Sex**	** **	** **	** **
Male	29.1 (27.2–31)	50.5 (48.7–52.2)	20.5 (18.8–22.2)
Female	36 (33.3–38.8)	39.5 (37–42.2)	24.4 (22–27.1)
**Height level of education**	** **	** **	** **
No education, preschool	40.2 (37.9–42.6)	46.5 (44.2–48.8)	13.3 (11.7–15.1)
Primary	28.1 (25.4–31)	50.8 (47.9–53.8)	21.1 (18.7–23.6)
Secondary	22.9 (19.8–26.4)	47.5 (44–51.1)	29.5 (26.3–33)
College or higher	6.5 (4.4–9.3)	39.0 (34.5–43.8)	54.5 (49.7–59.3)
**Currently working**	** **	** **	** **
No	37.1 (34.7–39.7)	40.6 (38.3–42.9)	22.3 (20.1–24.6)
Yes	27.4 (25.5–29.4)	51.2 (49.4–53.1)	21.3 (19.6–23.2)
**Wealth index**	** **	** **	** **
Poorest	46.5 (43.2–49.8)	46.9 (43.7–50)	6.6 (5.2–8.5)
Poorer	39.8 (36.5–43.3)	49.7 (46.1–53.3)	10.4 (8.5–12.7)
Middle	34.4 (31.2–37.8)	51.0 (47.8–54.2)	14.6 (12.3–17.2)
Richer	23.2 (20.4–26.2)	49.0 (45.6–52.5)	27.8 (24.7–31.1)
Richest	12.9 (10.7–15.4)	39.2 (36.3–42.2)	47.9 (44.9–51)
**Region**	** **	** **	** **
Barisal	36.6 (32.5–40.9)	48.7 (44.9–52.5)	14.8 (11.8–18.3)
Chittagong	30.6 (27.2–34.2)	44.8 (41.1–48.5)	24.6 (21.1–28.5)
Dhaka	29.8 (26.1–33.8)	48 (45–51)	22.2 (18.7–26.2)
Khulna	27.2 (23.7–30.9)	48 (44.9–51.1)	24.9 (21.6–28.4)
Rajshahi	29.5 (25.3–34.1)	47 (43–51.1)	23.5 (19.7–27.8)
Rangpur	36.9 (32.9–41)	48.7 (44.5–52.8)	14.4 (11.7–17.7)
Sylhet	35.8 (31.1–40.8)	43.0 (38.6–47.4)	21.2 (17.1–26)
**Place of residence**	** **	** **	** **
Urban	18.8 (16.2–21.8)	43.7 (40.8–46.6)	37.4 (34.2–40.8)
Rural	34.8 (32.9–36.7)	48.1 (46.4–49.9)	17.1 (15.5–18.8)

**Fig 2:**
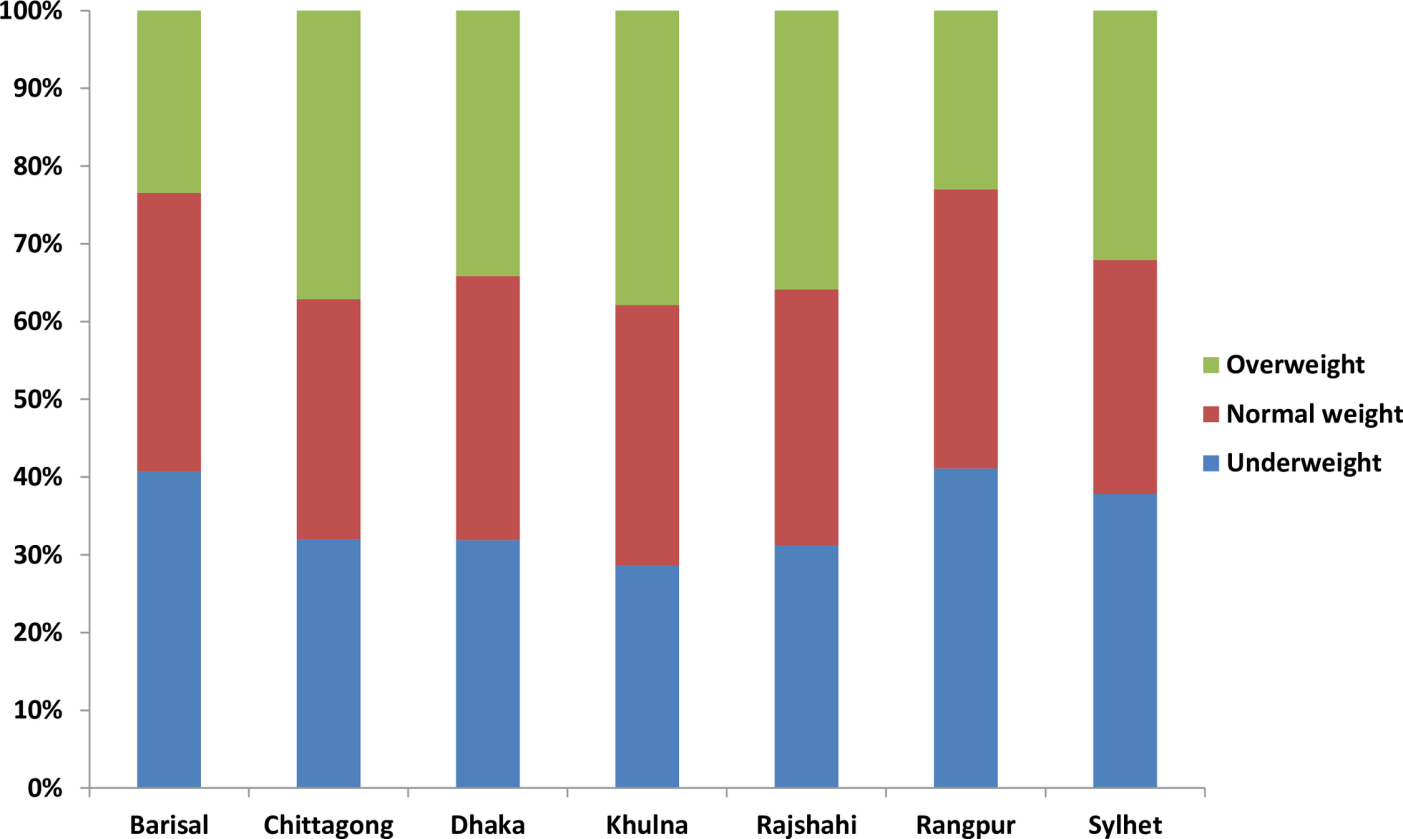
Nutritional statuses of adults living in the seven administrative divisions of Bangladesh.

**Fig 3:**
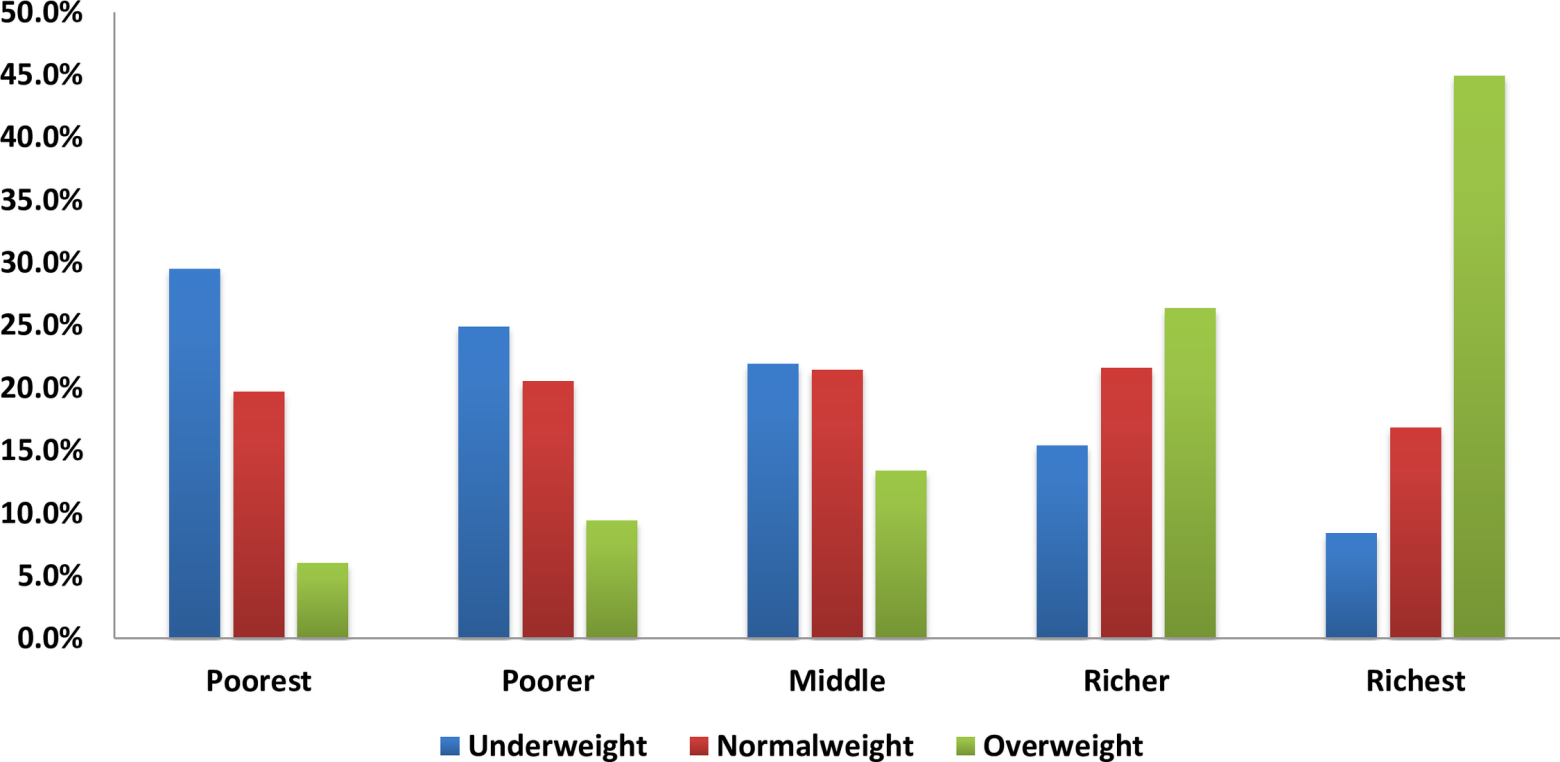
Nutritional status stratified by wealth index quintile.

The mean (± SD) age was 54.9±12.9 years, almost 69% were male and nearly half (48%) had no education. Most of the participants (61.4%) were in paid employment. Over three quarters of the participants (77.4%) lived in rural areas. Overall, 1668 people (30.4%, 95%CI: 29.2–31.6) were underweight, 1038 (18.9%, 95%CI: 17.9–19.9) were overweight and 253 (4.6%, 95%CI: 4.0–5.2) were obese. The prevalence of underweight for males and females were 29.1% and 36.0%, respectively, overweight was 17.4% and 18.4% and obesity was 3.0% and 6.0%, respectively. The nutritional status of the seven administrative divisions of Bangladesh was similar, Fig 2. However, large disparities in nutritional status were observed when stratified by wealth index, Fig 3.

### Determinants of underweight

Being underweight was significantly associated with age, sex, education, being involved in paid work, wealth and place of residence (urban vs. rural), Table 2. After adjusting for sex, education, employment, wealth, and place of residence, older people (≥70 years of age) were more than twice as likely (odds ratio (OR): 2.51, 95%CI: 1.95–3.23, p<0.001) to be underweight than the younger people (35 to 44 years). Similarly, those who had no education were three times more likely (OR: 3.59, 95%CI: 2.30–5.61, p<0.001) to be underweight compared to those with a college or higher education. The poorest quintile individuals were almost four times more likely (OR: 3.70, 95%CI 2.76–4.96, p<0.001) to be underweight compared to their wealthier counterparts. No difference was observed in terms of place of residence (urban vs rural) after adjusting for age, sex, education, employment and wealth.

**Table 2:** Unadjusted and adjusted odds ratios for factors associated with underweight compared to normal weight.

Variables	Unadjusted		Adjusted#	
	OR (95%CI)	p-value	OR (95%CI)	p-value
Age group (years)				
35–44	**Ref**		**Ref**	
45–54	1.53 (1.24–1.89)	<0.001	1.42 (1.14–1.77)	<0.001
55–69	1.84 (1.52–2.22)	<0.001	1.57 (1.27–1.94)	<0.001
70+	3.21 (2.62–3.93)	<0.001	2.51 (1.95–3.23)	<0.001
**Sex**				
Male	**Ref**		**Ref**	
Female	1.45 (1.27–1.66)	<0.001	0.87 (0.69–1.10)	0.24
**Educational status**				
No education, preschool	8.86 (5.88–13.36)	<0.001	3.59 (2.3–5.61)	<0.001
Primary	5.27 (3.45–8.05)	<0.001	2.75 (1.77–4.29)	<0.001
Secondary	4.07 (2.70–6.12)	<0.001	2.76 (1.82–4.20)	<0.001
College or higher	**Ref**		**Ref**	
**Currently working**				
No	1.61 (1.42–1.83)	<0.001	1.25 (0.98–1.60)	0.08
Yes	**Ref**		**Ref**	
**Wealth index**				
Poorest	5.20 (4.03–6.7)	<0.001	3.70 (2.76–4.96)	<0.001
Poorer	3.98 (3.09–5.12)	<0.001	2.89 (2.16–3.87)	<0.001
Middle	3.18 (2.46–4.11)	<0.001	2.40 (1.80–3.21)	<0.001
Richer	1.90 (1.46–2.48)	<0.001	1.58 (1.18–2.1)	<0.001
Richest	**Ref**		**Ref**	
**Region**				
Barisal	1.03 (0.78–1.35)	0.86	0.96 (0.74–1.26)	0.79
Chittagong	0.82 (0.63–1.07)	0.14	0.84 (0.65–1.08)	0.17
Dhaka	0.77 (0.58–1.01)	0.06	0.82 (0.64–1.05)	0.11
Khulna	0.67 (0.51–0.88)	<0.001	0.71 (0.54–0.92)	0.01
Rajshahi	0.77 (0.57–1.03)	0.08	0.78 (0.59–1.02)	0.07
Rangpur	1.02 (0.78–1.33)	0.90	0.9 (0.70–1.16)	0.40
Sylhet	**Ref**		**Ref**	
**Place of residence**				
Urban	**Ref**		**Ref**	
Rural	2.16 (1.76–2.64)	<0.001	1.15 (0.93–1.42)	0.19

#Adjusted odds ratios have been adjusted for all variables listed

### Determinants of overweight and obesity

Being overweight or obese was also associated with age, sex, education, wealth and place of residence, Table 3. In contrast to underweight, younger age (35–44 years), being female, having some education was associated with overweight/obesity compared to those with no education, as was being wealthier and living in urban areas. Females were more than twice as likely to be overweight/obese compared to males (OR: 2.48, 95%CI: 1.87–3.28, p<0.001). Those with a college or higher education were almost four times more likely (OR: 3.98, 95%CI: 2.96–5.33, p<0.001) to be overweight/obese compared to those with no education; wealthy individuals were seven times more likely than their poorer counterparts (OR: 7.14, 95%CI: 5.20–9.8, p<0.001). There was also significant difference in the prevalence of overweight/obesity by place of residence; those living in urban areas were more likely to be overweight/obese compared to participants living in rural areas (OR: 1.27, 95%CI: 1.05–1.55, p = 0.02).

**Table 3:** Unadjusted and adjusted odds ratios for factors associated with overweight/obesity compared to normal weight.

Variables	Unadjusted		Adjusted#	
	OR (95%CI)	p-value	OR (95%CI)	p-value
**Age group** (years)				
35–44	1.90 (1.49–2.42)	<0.001	1.73 (1.24–2.41)	<0.001
45–54	1.80 (1.43–2.28)	<0.001	1.61 (1.2–2.17)	<0.001
55–69	1.46 (1.13–1.87)	<0.001	1.19 (0.89–1.57)	0.24
70+	**Ref**		**Ref**	
**Sex**				
Male				
Female	1.26 (1.09–1.45)	<0.001	2.48 (1.87–3.28)	<0.001
**Educational status**				
No education, preschool	**Ref**		**Ref**	
Primary	1.74 (1.45–2.10)	<0.001	1.54 (1.23–1.92)	<0.001
Secondary	2.73 (2.23–3.34)	<0.001	1.93 (1.49–2.50)	<0.001
College or higher	7.67 (6.07–9.70)	<0.001	3.98 (2.96–5.33)	<0.001
**Currently working**				
No	1.06 (0.92–1.22)	0.42	0.91 (0.70–1.20)	0.51
Yes	**Ref**		**Ref**	
**Wealth index**				
Poorest	**Ref**		**Ref**	
Poorer	1.66 (1.17–2.36)	<0.001	1.52 (1.07–2.17)	<0.001
Middle	2.43 (1.74–3.39)	<0.001	1.98 (1.40–2.80)	<0.001
Richer	5.42 (3.97–7.40)	<0.001	3.79 (2.74–5.24)	<0.001
Richest	13.12 (9.84–17.50)	<0.001	7.14 (5.20–9.81)	<0.001
**Region**				
Barisal	0.64 (0.44–0.92)	0.02	0.72 (0.52–0.99)	0.05
Chittagong	1.23 (0.89–1.7)	0.22	1.13 (0.84–1.52)	0.42
Dhaka	1.06 (0.76–1.49)	0.72	0.86 (0.64–1.16)	0.32
Khulna	1.24 (0.91–1.70)	0.18	1.2 (0.89–1.61)	0.22
Rajshahi	1.15 (0.82–1.62)	0.42	1.24 (0.91–1.69)	0.18
Rangpur	0.63 (0.44–0.89)	0.01	0.84 (0.61–1.16)	0.28
Sylhet	**Ref**		**Ref**	
**Place of residence**				
Urban	2.92 (2.44–3.5)	<0.001	1.27 (1.05–1.55)	0.02
Rural	**Ref**		**Ref**	

#Adjusted odds ratios have been adjusted for all variables listed.

## Discussion

This is the first study to report the prevalence of underweight and overweight/obesity, using Asian specific BMI cut-offs, determined by measured height and weight, in a nationally representative group of Bangladeshi adults aged ≥35 years. The results demonstrate the co-existence of dual burden of underweight and overweight/obesity. The overall prevalence of underweight (30%) was still higher that the prevalence of overweight/obesity (24%). However, the prevalence of overweight/obesity was higher than underweight in the younger age groups potentially indicating an advancing wave of obesity. Similar to previous studies in developing countries living in a rural environment with little education and low wealth index was associated with greater under nutrition and living in an urban environment and a higher socioeconomic status was associated with overweight/obesity [1].

There is a paucity of national published data examining the prevalence of overweight and obesity in the population of Bangladesh. The prevalence of overweight and obesity that we report in urban Bangladesh (37%) is double the previous report of 17% based on a hospital (icddr,b) population of adults (≥20 years of age) in 2011 [18]. We found the prevalence of overweight and obesity in the rural areas (17%) was also higher than the 10% reported in rural women of reproductive age in 2000–2004 [19], but was less than half the prevalence reported in a rural community (aged ≥20 years) in 2009 which indicated that ~18% were overweight and 26% were obese[20]. In part, the discrepancy is due to different cut-points used to define overweight and obesity, as well as differences in the age of the population studied. We noted that the prevalence of overweight/obesity increased with decreasing age with the younger group (35 to 44 years) almost twice as likely to be overweight/obese compared to the older group (≥ 70 years), but there was little difference in the proportion of overweight/obese females compared to males, 22% and 20%, respectively. Nevertheless, there is little doubt that there has been a substantial increase in overweight/obesity from the early 1990s when the prevalence of overweight and obesity was reported to be 4% [18]. We speculate that the increasing prevalence of overweight/obesity is the principal driver for the 2.5 fold increase in diabetes in Bangladesh from 4% in 1990 to 10% in 2011[21] and will lead to further increases in morbidity and mortality from chronic disease conditions including hypertension, cardiovascular disease and cancer.

We also report that almost one in three Bangladeshi adults aged ≥35 years was underweight. Similar to overweight/obesity, there is a paucity of comparative figures for the general population, particularly males. For females, the prevalence that we report (36%) is similar to the 33% of ever-married women aged 15 to 49 years in 2004 but greater than the 24% of ever-married women of the same age in 2011[18]. The difference is likely to reflect the different age of the population, the proportion individuals who were underweight increased with older age. However, there is also a large wealth disparity; approximately 8% of the richest (highest wealth index) adults were underweight and 30% with the lowest wealth index; more than a threefold increase in underweight, Fig 3. The wealth disparity is comparable to the 2007 findings in ever married women; 13% of the richest quintile were underweight compared to 42% of the poorest quintile[22]. It is well recognized that underweight in women of childbearing age is a risk factor for adverse pregnancy outcomes, such as intrauterine growth retardation and low birth weight infants[23]. In addition to females, one in four (28%) males was also underweight. Remarkably we could not find any previously published comparative Bangladeshi data, despite the inverse associations between parental BMI and childhood undernutrition, of a similar strength to maternal BMI, in India[24] and the positive association between adult BMI and reduced performance capacity and low productivity [3]. One explanation for the lack of data may be that the prevalence of under nutrition in women is considered to be a proxy for under nutrition prevalence in all adults [3].

The strength of this study is that it is analysis of a large national sample consisting of both urban and rural populations in Bangladesh. However, there are a number of limitations. While the study was reported to be representative, only two thirds of the target age group (≥35 years) had anthropometry measured. However, because of the sampling strategy used we believe the sample is likely to be representative of the population. It is also clear that females are underrepresented; ie 30% of the study population but make up ~49% of the total Bangladeshi population [15]. The implications of this are not clear. Thirdly, the survey is cross-sectional and the analyses presented are associations and causality between nutritional status and determinants cannot be elucidated. In addition, the BDHS 2011, did not include information on dietary habits or physical activity and hence major determinants of nutritional status were not explored. Finally, nutrition status was determined by BMI which does not differentiate between fat and fat free mass. However, BMI is the most common anthropometric measure used to determine adiposity and a recent study has demonstrated a strong correlation (r >0.8) with the mid-upper arm circumference in underweight Bangladeshi adults[25] and the association between high BMI and with non-communicable disease has been well described[26].

## Conclusion

In summary, these data indicate the co-existence of the dual burden of underweight and overweight/obesity in Bangladeshi adult’s ≥35 years of age. There is strong evidence to suggest that the proportion of adults categorized as overweight and obesity has increase substantially over the last two decades, particularly amongst the wealthiest, most educated, living in urban areas. The increased prevalence of overweight/obese in the younger adults also indicates that the problem of overweight/obesity, in the absence of effective treatment, is likely to get worse overtime. Nevertheless, underweight still remains a major public health issue for both females and males effecting ~30% of the population ≥35 years of age and particularly the poor. Both conditions are associated with increased risk of morbidity and mortality including risk of developing non-communicable diseases. Public health interventions to address both conditions and associated risk factors are essential for the health of the people Bangladesh.
